# Crystal Structures of the Carborane Dianions [1,4-(PhCB_10_H_10_C)_2_C_6_H_4_]^2−^ and [1,4-(PhCB_10_H_10_C)_2_C_6_F_4_]^2−^ and the Stabilizing Role of the *para*-Phenylene Unit on 2 *n*+3 Skeletal Electron Clusters**

**DOI:** 10.1002/anie.201310718

**Published:** 2014-02-26

**Authors:** Jan Kahlert, Hans-Georg Stammler, Beate Neumann, Rachel A Harder, Lothar Weber, Mark A Fox

**Keywords:** carborane, conjugation, electrochemistry, phenylene, quinoidal structures

## Abstract

While carboranes with 2 *n*+2 and 2 *n*+4 (*n*=number of skeletal atoms) skeletal electrons (SE) are widely known, little has been reported on carboranes with odd SE numbers. Electrochemical measurements on two-cage assemblies, where two C-phenyl-*ortho*-carboranyl groups are linked by a *para*-phenylene or a *para*-tetrafluorophenylene bridge, revealed two well separated and reversible two-electron reduction waves indicating formation of stable dianions and tetraanions. The salts of the dianions were isolated by reduction with sodium metal and their unusual structures were determined by X-ray crystallography. The diamagnetic dianions contain two 2 *n*+3 SE clusters where each cluster has a notably long carborane C–carborane C distance of ca 2.4 Å. The π conjugation within the phenylene bridge plays an important role in the stabilization of these carboranes with odd SE counts.

Icosahedral dicarba-*closo*-dodecaboranes, also known as carboranes, have fascinated researchers due to their unique properties such as high thermal stability, spherical shape, and three-dimensional delocalization of σ-framework electrons.^1^, ^2^ They are currently explored preferentially in the fields of medicinal chemistry^3^ and luminescent materials.^4^ Electron-counting rules dictate that these *closo*-clusters are held together by 2 *n*+2 skeletal electrons (SE) where *n* represents the number of skeletal atoms.^5^ Addition of two electrons to *closo*-carboranes by reduction with alkali metals leads to more open *nido* geometries with a 2 *n*+4 skeletal electron count.^6^, ^7^ While *closo*- and *nido*-carboranes are widely known, little is reported^8^, ^9^ on carboranes with odd numbers of skeletal electrons and no crystallographic studies are published on carborane radical anions from 12-vertex carboranes.

Diphenyl-*ortho*-carborane, 1,2-Ph_2_-1,2-C_2_B_10_H_10_ (**1**; Scheme 1), shows two one-electron reduction waves indicating the presence of a stable radical anion [**1**]^−^ with a 2 *n*+3 SE count.^10^ Spectroelectrochemical experiments, EPR measurements and calculations on the radical anion [**1**]^−^ suggest that the free electron is delocalized within the carborane cluster and the carborane C1═C2 bond is lengthened to 2.39 Å in [**1**]^−^ compared to 1.76 Å in **1** at B3LYP/6-31G*.^11^ The phenyl groups remain little changed on going from **1** to [**1**]^−^.

**Scheme 1:**
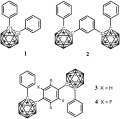
Diphenyl-*ortho*-carborane 1 and the two-cluster assemblies 2, 3 and 4.

The C1═C2 bond in *ortho*-carborane is well known for its elasticity.^1^, ^12^ 1,2-C_2_B_10_H_12_ itself has a C═C bond length of 1.62 Å^13^ which can be elongated up to 2.15 Å^14^ with bulky substituents at both C1 and C2 and up to 2.42 Å^6^ by substituents capable of forming multiple bonds with a carborane C atom. Recently, molecules containing the diphenyl-*ortho*-carborane unit were reported to have remarkable luminescence properties with the excited electron being transferred from the aromatic ring to the cluster after excitation.^15^ It is assumed that the excited-state geometries in diaryl-*ortho*-carboranes contain long C1═C2 distances like the geometries of the corresponding radical anions.^16^, ^17^

Given that a closer look at the unusual 2 *n*+3 SE anions can improve our understanding of the luminescence properties of diaryl-*ortho*-carboranes, we decided to examine the reduction properties of the two-cage assemblies, **2**, **3** and **4**, where two C-phenyl-*ortho*-carboranyl groups are linked by a *meta*-phenylene, a *para*-phenylene and a *para*-tetrafluorophenylene bridge, respectively (Scheme 1).^18^, ^19^ If these bridges act as insulating spacers, reductions of **2**–**4** should produce dianions [**2**–**4**]^2−^ with two 2 *n*+3 SE clusters and then tetraanions [**2**–**4**]^4−^ at similar potentials as those of **1** in the CV experiment (Table 1).

**Table 1:** Cyclic voltammetry data for the observed reduction waves of 1–4.

	*E*_1/2_ (Red1) [V]	*E*_1/2_ (Red2) [V]	*E*_1/2_ (Red3) [V]	*E*_1/2_ (Red4) [V]
1e waves	0/−1	−1/−2	−2/−3	−3/−4
**1**	−1.69	−1.85		
**2**	−1.60	−1.75	−1.86	−1.95
				
2e waves	0/−2	−2/−4		
**3**	−1.56	−1.94		
**4**	−1.20	−1.79		

The cyclic voltammogram for the 1,3-bis-(2′-phenyl-*ortho*-carboran-1′-yl)-benzene **2** originates from four overlapping reversible one-electron reduction waves (Figure S12 in the Supporting Information) which was confirmed by square wave voltammetry where all four reduction events are unequivocally resolved (Figure S13 and Table S2).^20^ Thus, the mono-, di-, tri- and tetraanions of **2** are formed in a stepwise manner on reduction. By contrast, cyclic voltammograms of the two 1,4-bis-(2′-phenyl-*ortho*-carboran-1′-yl)-benzenes **3** and **4** display two well-defined two-electron reversible reduction waves (Figure S12 and Table 1) with peak separations of 380 and 590 mV, respectively. After reduction at the first wave potential, a deep purple color was observed in solution at the glassy carbon electrode surface. The color changed to yellow after the second reduction wave for compounds **3** and **4**. As the peak separations are large for **3** and **4**, we decided to isolate the blue reduced species by chemical reductions to establish whether the phenylene bridges play important roles.

A deep blue color appeared when solutions of **3** or **4** in dimethoxyethane (DME) were sonicated for 2.5 h in the presence of an excess of sodium. The blue diamagnetic salts ([Na(dme)_3_]^+^)_2_[**3**]^2−^ and ([Na(dme)_3_]^+^)_2_[**4**]^2−^ were isolated by crystallization in 52 and 61 % yield, respectively (for detailed synthetic procedures see the Supporting Information). Blue solutions of the products in tetrahydrofuran (THF), DME, acetonitrile and dichloromethane were stable under an atmosphere of argon or dinitrogen. NMR spectra (Figures S1–S11) confirm their diamagnetic nature. The simple peak pattern in the range of −6 to −30 ppm in the ^11^B{^1^H} NMR spectrum of [**3**]^2−^ suggests that both clusters are identical and thus reduction takes place involving both clusters.

Contact of the solutions or the solid salts with air led to the complete loss of the color and the formation of the neutral precursors quantitatively. When the sodium metal reactions of **3** and **4** are sonicated for over 24 h, these blue solutions turned yellow/orange indicating the formation of tetraanions as expected from the strong reducing power of the sodium metal. Clearly, the isolated dianions [**3**]^2−^ and [**4**]^2−^ correspond to the products of only the first reduction waves in the cyclic voltammograms of **3** and **4**.

Single crystals of the salts ([Na(dme)_3_]^+^)_2_[**3**]^2−^ and ([Na(dme)_3_]^+^)_2_[**4**]^2−^ suitable for X-ray crystallography were obtained from DME solutions at low temperatures and measured at 100(2) K (Figure 1).^20^ In both structures, a crystallographic inversion center is located in the center of the bridging arene ring and the terminal phenyl groups point to opposite sides of the plane defined by the central ring. Table 2 lists important bond lengths of the dianions [**3**]^2−^ and [**4**]^2−^ with corresponding bond lengths of the neutral species **3** and **4** included for comparison. Scheme 2 shows the atom numbering used here.

**Figure 1:**
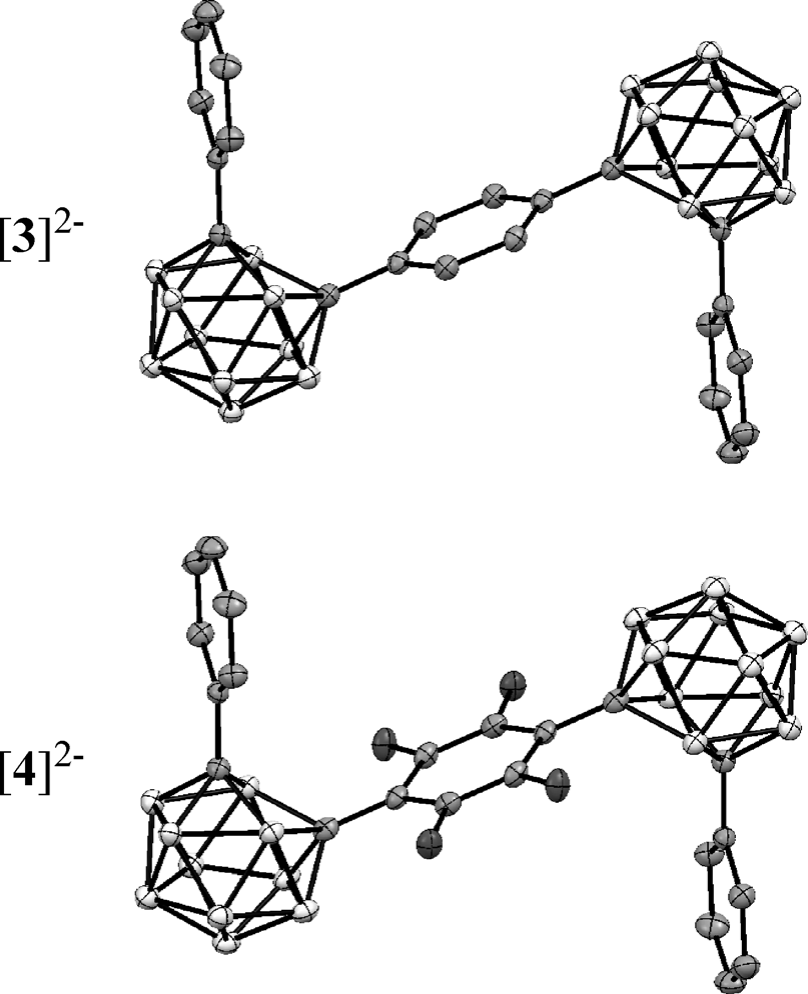
Molecular structures of the dianions [3]^2−^ and [4]^2−^ in the crystals. The tris(dimethoxyethane)sodium cations and hydrogen atoms are omitted for clarity.

**Scheme 2:**
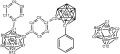
Generic structures for [3]^2−^ and [4]^2−^ (left) and 5 (right) with atom numbering. The Me_3_Si groups attached to the carbon atoms in 5 are omitted for clarity.

**Table 2:** Selected bond lengths of 3, 4, their dianions [3]^2−^ and [4]^2−^ and compound 5 determined by X-ray crystallography.

	3	[3]^2−^	4	[4]^2−^	5
C1-C2	1.719(2)	2.370(1)	1.732(2)	2.387(2)	2.504(3)
C1-B3/B6	1.731(2)/1.732(2)	1.694(1)/1.703(1)	1.731(3)/1.730(3)	1.690(2)/1.696(2)	1.672(3)/1.685(3)
C2-B3/B6	1.735(2)/1.736(2)	1.786(1)/1.775(1)	1.742(3)/1.745(3)	1.820(2)/1.815(2)	1.682(3)/1.670(3)
C1-B4/B5	1.714(2)/1.714(2)	1.646(1)/1.647(1)	1.711(3)/1.714(3)	1.649(2)/1.650(2)	1.604(3)/1.596(3)
C2-B7/B11	1.718(2)/1.719(2)	1.632(1)/1.635(1)	1.696(3)/1.707(3)	1.631(2)/1.635(2)	1.601(3)/1.585(3)
C1-C6	1.508(2)	1.477(1)	1.515(2)	1.484(2)	
C2-C3	1.506(2)	1.424(1)	1.512(2)	1.421(2)	
C3-C4/C3-C5	1.386(2)/1.393(2)	1.419(1)/1.421(1)	1.388(3)/1.392(3)	1.424(2)/1.422(2)	
C4-C5′	1.389(3)	1.368(1)	1.375(2)	1.359(2)	

The most remarkable features of both structures are the C1═C2 distances for [**3**]^2−^ and [**4**]^2−^ which are lengthened by 0.65 Å and 0.66 Å, respectively, in comparison to their neutral precursors. Changes in the bonds C1═B3/6 and C1═B4/5 compared to changes in C2═B3/6 and C2═B7/11 in Table 2 reveal that the C2 atom has been shifted more away from its original position in the cluster scaffold upon reduction than the C1 atom.

As the unusual cuboctahedron carborane geometry of (Me_3_Si)_4_C_4_B_8_H_8_ **5** also contains rectangular C_2_B_2_ faces,^21^ this 2 *n*+4 SE *nido*-geometry determined experimentally is compared with the 2 *n*+3 clusters of the dianions [**3**]^2−^ and [**4**]^2−^ as listed in Table 2. The C_2_B_2_ faces in **5** are more regular than those in the dianions reflecting the lower cage symmetry in the dianions caused by different interactions with C_6_H_5_ rings and the C_6_X_4_ bridges.

While both clusters in these systems **3** and **4** are significantly distorted on reduction, the phenyl groups remain largely unchanged whereas the ═C_6_H_4_═ and ═C_6_F_4_═ bridges are distorted as well. The bridges become more quinoid-like with the bond between the aryl ring and the cluster shortened by 0.08–0.09 Å. While this bond shortening agrees with an increased bond order, it is quite small when compared to carborane anions with substantial *exo*-multiple bond character.^6^, ^12^ The geometrical parameters of the ═C_6_H_4_═ ring in [**3**]^2−^ resemble the phenylene units in the experimentally determined geometries of *para*-quinodimethane analogues, disilaquinodimethane **6** and diboraquinodimethane **7** (Scheme 3).^22^, ^23^ The transitions responsible for the colors in [**3**]^2−^ (686 nm), **6** (555 nm) and **7** (630 nm) presumably arise from the ═C_6_H_4_═ ring in all cases.

**Scheme 3:**
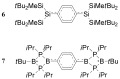
Structures of disilaquinodimethane 6 and diboraquinodimethane 7.

In order to determine whether each cluster in the dianions contains 2 *n*+3 skeletal electrons, natural population analyses (NPA) were carried out on computed geometries of **3**, [**3**]^2−^, **4** and [**4**]^2−^ and compared with data for **1** and [**1**]^−^ (Table S4). The optimized geometries of [**3**]^2−^ and [**4**]^2−^ at B3LYP/6-31G* in the diamagnetic states agree with the X-ray crystallographic data and computed ^11^B and ^13^C GIAO-NMR chemical shifts from the optimized geometries also fit well with observed NMR peak shifts (Table S5 and Figure S15). The C1═C2 distances, Wiberg bond indices (BI) and charges on the clusters of the anions [**1**]^−^, [**3**]^2−^ and [**4**]^2−^ are all very similar which suggest that each cluster in the dianions has a formal 2 *n*+3 SE count and a −1 charge. The 1,4-C_6_X_4_ rings are virtually neutral in [**3**]^2−^ and [**4**]^2−^ with computed charges close to zero and the bond orders of the rings reflect their quinoid-like character where all three bonds at C3 have similar bond orders of 1.22–1.24 and the C4═C5′/C5═C4′ bond orders are 1.51–1.60.

The *meta*-phenylene bridge in **2** transmits electronic effects inductively since the electrochemical reduction results in four observed waves close together and at slightly lower potentials than **1** (Table 1). The potential for the two-electron reduction is lowered by 0.19 V for the *para*-phenylene analogue **3** to [**3**]^2−^ compared to **2** to [**2**]^2−^. The significant structural rearrangement in the formation of [**3**]^2−^ causes a shift in the standard potentials of the two one electron-transfer reactions to promote the second electron transfer and produce the 2-electron wave observed. The second electron transfer is thus thermodynamically easier than the first. The increased stability of the 2 *n*+3 clusters in [**3**]^2−^ compared to [**2**]^2−^ is thus attributed to the unique property of the 1,4-C_6_H_4_ unit.

There has to be some degree of conjugation in [**3**]^2−^ (and [**4**]^2−^) that stabilizes these 2 *n*+3 clusters. Thus, the orientation of the phenylene ring with respect to one cluster in [**3**]^2−^ was explored computationally. Constraining the phenylene ring in plane with the C1═C2 axis gave a geometry still retaining the quinoid-like ring but the C2═C3 bond is lengthened at 1.483 Å and C2′═C3′ at 1.457 Å compared to the C2/C2′═C3/C3′ bonds of 1.421 Å in the fully optimized geometry [**3**]^2−^. The energy of the constrained geometry is 17.3 kcal mol^−1^ higher than the energy of the fully optimized geometry. Such a difference in energy indicates that there is a strong orientational preference in the conjugation between the ring and the cluster. By contrast, the energy difference for the two phenyl group orientations in the radical anion [**1**]^−^ is only 2.7 kcal mol^−1^—a value close to those found for neutral C-aryl *ortho*-carboranes.^24^ The conjugations in the dianions [**3**]^2−^ and [**4**]^2−^ presumably are favorable interactions between the aromatic π orbitals at the 1,4-C_6_H_4_ bridge with the cluster tangent p-orbitals aligned along the C1═C2 axis at C2/C2′. The increased stability of the dianion [**3**]^2−^ compared to [**2**]^2−^ is a result of π conjugation between the ring and cluster.

In conclusion, reductions of compounds with C-aryl-*ortho*-carborane groups substituted at the *para* positions of a benzene ring give dianions [**3**]^2−^ and [**4**]^2−^ where each carborane cluster has a rare 2 *n*+3 SE count. These dianions are stabilized by π conjugation with the *para*-phenylene unit which adopts a geometry that is intermediate between a quinoid system and an aromatic ring. There are many assemblies in the literature^1^ with two or more carboranyl groups directly attached to an aromatic or heteroaromatic ring so reductions of these systems may generate anions with 2 *n*+3 clusters that can be isolated if the ring-cluster link is capable of π conjugation like in [**3**]^2−^ and [**4**]^2−^. This conjugation has implications in designing extended π-bond systems involving C-aryl carboranes and indeed in the intriguing fluorescence properties of C-aryl carboranes.

## Supporting Information

As a service to our authors and readers, this journal provides supporting information supplied by the authors. Such materials are peer reviewed and may be re-organized for online delivery, but are not copy-edited or typeset. Technical support issues arising from supporting information (other than missing files) should be addressed to the authors.
