# Supplementary material for: Crystal Structures of the Carborane Dianions [1,4-(PhCB10H10C)2C6H4]2− and [1,4-(PhCB10H10C)2C6F4]2− and the Stabilizing Role of the para-Phenylene Unit on 2 n+3 Skeletal Electron Clusters
Source: Angew Chem Int Ed Engl. 2014 Feb 26;53(14):3702–5. doi: 10.1002/anie.201310718 (PMC4257503; doi:10.1002/anie.201310718)
Supplement: Supplementary file 1 [file anie0053-3702-SD1.pdf]

Angewandte Chemie
Eine Zeitschrift der Gesellschaft Deutscher Chemiker

Supporting Information

© Wiley-VCH 2014

69451 Weinheim, Germany

# Crystal Structures of the Carborane Dianions $[1,4\text{-}(PhCB_{10}H_{10}C)_2C_6H_4]^{2-}$ and $[1,4\text{-}(PhCB_{10}H_{10}C)_2C_6F_4]^{2-}$ and the Stabilizing Role of the *para*-Phenylene Unit on $2n+3$ Skeletal Electron Clusters**

*Jan Kahlert, Hans-Georg Stammler, Beate Neumann, Rachel A. Harder, Lothar Weber,* and Mark A. Fox**

anie_201310718_sm_miscellaneous_information.pdf

**SUPPORTING INFORMATION**

## Contents


| | Page |
|---|---|
| General experimental details | S2 |
| Syntheses and spectroscopic characterization | S2-S3 |
| Figures of NMR spectra | S4-S9 |
| Electrochemistry details | S10-S12 |
| X-ray crystallography details | S13-S14 |
| Computational details and data | S15-S17 |
| Cartesian coordinates for [**3**]$^{2-}$ and [**4**]$^{2-}$ | S18-S20 |
| References | S21 |

## Experimental section

### General

Unless otherwise stated all manipulations were performed under strict exclusion of moisture and oxygen in an atmosphere of dry argon by using standard Schlenk techniques. Solvents were dried with the respective drying agent (potassium for THF; calcium hydride for DME, acetonitrile and dichloromethane; lithium aluminium hydride for *n*-hexane) and freshly distilled prior to use. 1,3-Bis-(2'-phenyl-*ortho*-carboran-1'-yl)-benzene (**2**), 1,4-bis-(2'-phenyl-*ortho*-carboran-1'-yl)-benzene (**3**) and 1,4-bis-(2'-phenyl-*ortho*-carboran-1'-yl)-2,3,5,6-tetrafluorobenzene (**4**) were prepared according to literature methods.[1,2] Sodium was purchased from commercial sources, freed from paraffin oil by washing with *n*-hexane and freshly cut prior to use. NMR spectra were recorded from solutions in $CD_3CN$ at room temperature on a Bruker AM Avance DRX500, a Bruker Avance III 500 and a Bruker Avance 600 spectrometer with $SiMe_4$ ($^1H$, $^{13}C$), $BF_3{\cdot}OEt_2$ ($^{11}B$) and $CFCl_3$ ($^{19}F$) as external standards. $^1H$- and $^{13}C\{^1H\}$ NMR spectra were calibrated on the solvent signal [1.94 ppm ($^1H$), 1.32 ppm ($^{13}C$)].

### Syntheses and spectroscopic characterization

Synthesis of $([Na(dme)_3]^+)_2[\mathbf{3}]^{2-}$:

Sodium (98 mg, 4.26 mmol) was added in one piece to a solution of 1,4-bis-(2´-phenyl-1´,2´-dicarbadodecaboran-1´-yl)-benzene (**3**) (0.25 g, 0.49 mmol) in DME (32 mL) and the mixture was sonicated for 2.5 h. The deep blue solution was filtered hot and the filtration residue was extracted with hot DME (5 mL). The combined filtrates were freed from solvent *in vacuo*. The remainder was recrystallized from DME (5 mL) and the crystals were washed with *n*-hexane (2 × 1 mL). The product was obtained as dark green solid. Yield: 0.28 g (52 %). Found: C, 49.65; H, 8.55 %; $C_{46}H_{94}O_{12}B_{20}Na_2$ requires C, 50.16; H, 8.60 %; $^1H$-NMR (500 MHz): δ [ppm] = 0.0 - 1.2 (m, br, 4 H, BH), 1.3 - 3.2 (m, br, 16 H, BH), 3.29 (s, 36 H, $CH_3$), 3.46 (s, 24 H, $CH_2$), 6.66 (s, br, 4 H, $H_{meta}$), 7.00 (s, br, 6 H, $C_6H_4$, $H_{para}$), 7.29 (s, br, 4H, $H_{ortho}$); $^1H\{^{11}B\}$-NMR (500 MHz): δ [ppm] = 0.44 (s, br, 2 H), 0.76 (s, br, 2 H), 2.12 (s, br, 12 H), 2.63 (s, br, 4 H); $^{13}C\{^1H\}$-NMR (600 MHz): δ [ppm] = 58.9 (s, $CH_3$), 66.2 (s, C2´), 72.3 (s, $CH_2$), 97.7 (s, C1´), 124.9 (s, $C_{meta}$), 127.2 (s, $C_{para}$), 128.2 (s, $C_{ortho}$), 129.5 (s, C2,3,5,6), 146.4 (s, C1,4), 147.9 (s, $C_{ipso, Ph}$); $^{11}B\{^1H\}$-NMR (500 MHz): δ [ppm] = –29.8 (2B, s), –11.8 (3B, s), –10.9 (2B, s), –8.3 (2B, s), –6.5 (1B, s).

Synthesis of $([Na(dme)_3]^+)_2[\mathbf{4}]^{2-}$:

A piece of sodium was added to a solution of 1,4-bis-(2'-phenyl-*ortho*-carboran-1'-yl)-2,3,5,6-tetrafluorobenzene (**4**) (0.22 g, 0.38 mmol) in DME (6 mL), whereupon a blue color occurred immediately at the sodium surface. The mixture was sonicated for 2.5 h and filtered hot subsequently. The filtration remainder was extracted with hot DME (2 × 5 mL) and the combined filtrates were freed from volatile materials *in vacuo*. The remainder was recrystallized from DME (4 mL) and the crystals were washed with *n*-hexane (2 × 1 mL). The product was obtained as dark blue solid. Yield: 0.27 g (61 %). $^1$H-NMR (500 MHz): δ [ppm] = 0.2 - 1.4 (m, br, 4 H, BH), 1.4 - 3.2 (m, br, 16 H, BH), 3.29 (s, 36 H, $CH_3$), 3.46 (s, 24 H, $CH_2$), 6.98 (m, 4 H, $H_{meta}$), 7.16 (t, $^3J_{HH}$ = 6.4 Hz, 2 H, $H_{para}$), 7.29 (d, $^3J_{HH}$ = 7.1 Hz, 4 H, $H_{ortho}$); $^1$H{$^{11}$B}-NMR (500 MHz): δ [ppm] = 0.64 (s, br, 2 H), 1.03 (s, br, 2 H), 2.02 (s, br, 4 H), 2.17 (s, br, 8 H), 2.71 (s, br, 4 H); $^{13}$C{$^1$H}-NMR (500 MHz): δ [ppm] = 58.9 (s, $CH_3$), 69.7 (s, C2´), 72.3 (s, $CH_2$), 100.2 (s, C1´), 122.2 (s, C1,4), 125.3 (s, $C_{para}$), 128.5 (s, $C_{meta}$), 129.2 (s, $C_{ortho}$), 142.6 (d, $^1J_{CF}$ = 237.0 Hz, C2,3,5,6), 146.8 (s, $C_{ipso, Ph}$); $^{11}$B{$^1$H}-NMR (500 MHz): δ [ppm] = –27.1 (2B, s), –11.2 (2B, s), –9.8 (3B, s), –7.4 (2B, s), –4.7 (1B, s); $^{19}$F-NMR (500 MHz): δ [ppm] = –146.7 (s).

**Figure S1:** $^{1}$H NMR spectrum of ([Na(dme)$_3$]$^+$)$_2$[**3**]$^{2-}$.

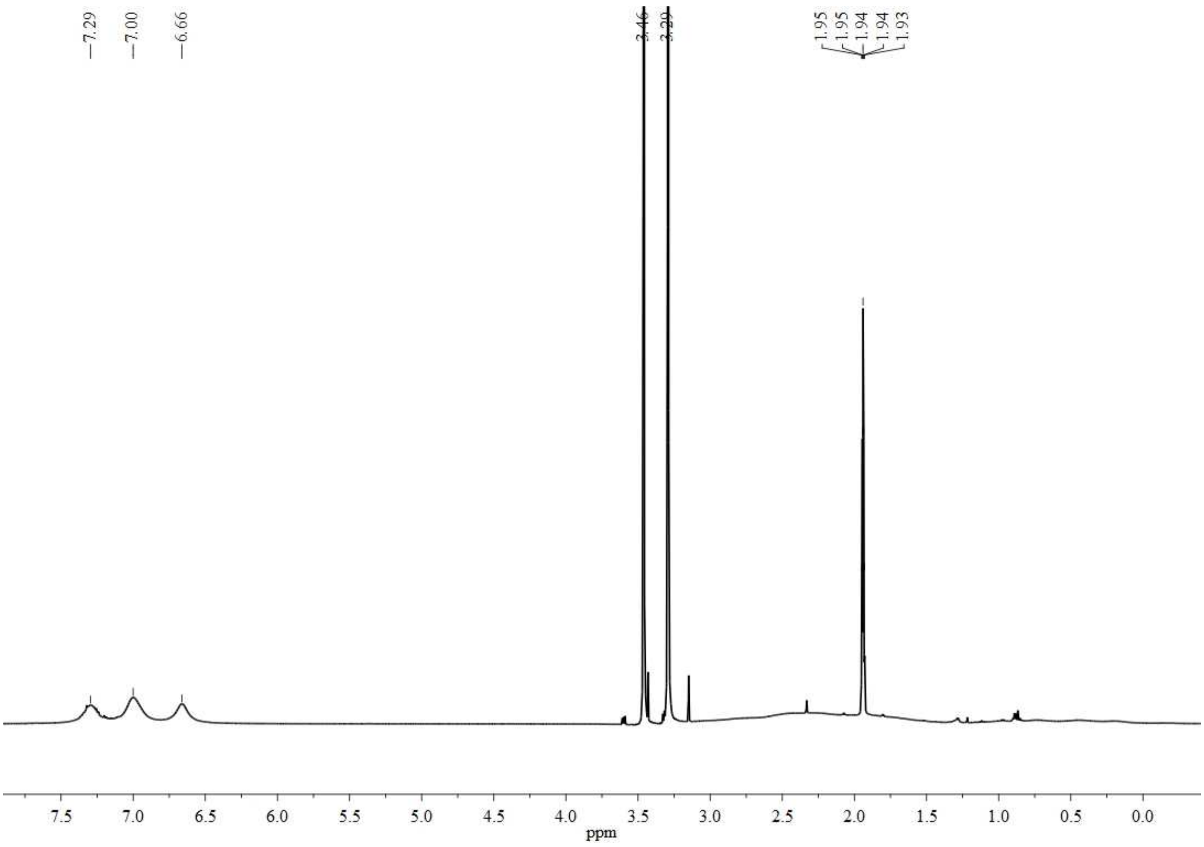


**Figure S2:** $^{1}$H{$^{11}$B} NMR spectrum of ([Na(dme)$_3$]$^+$)$_2$[**3**]$^{2-}$.

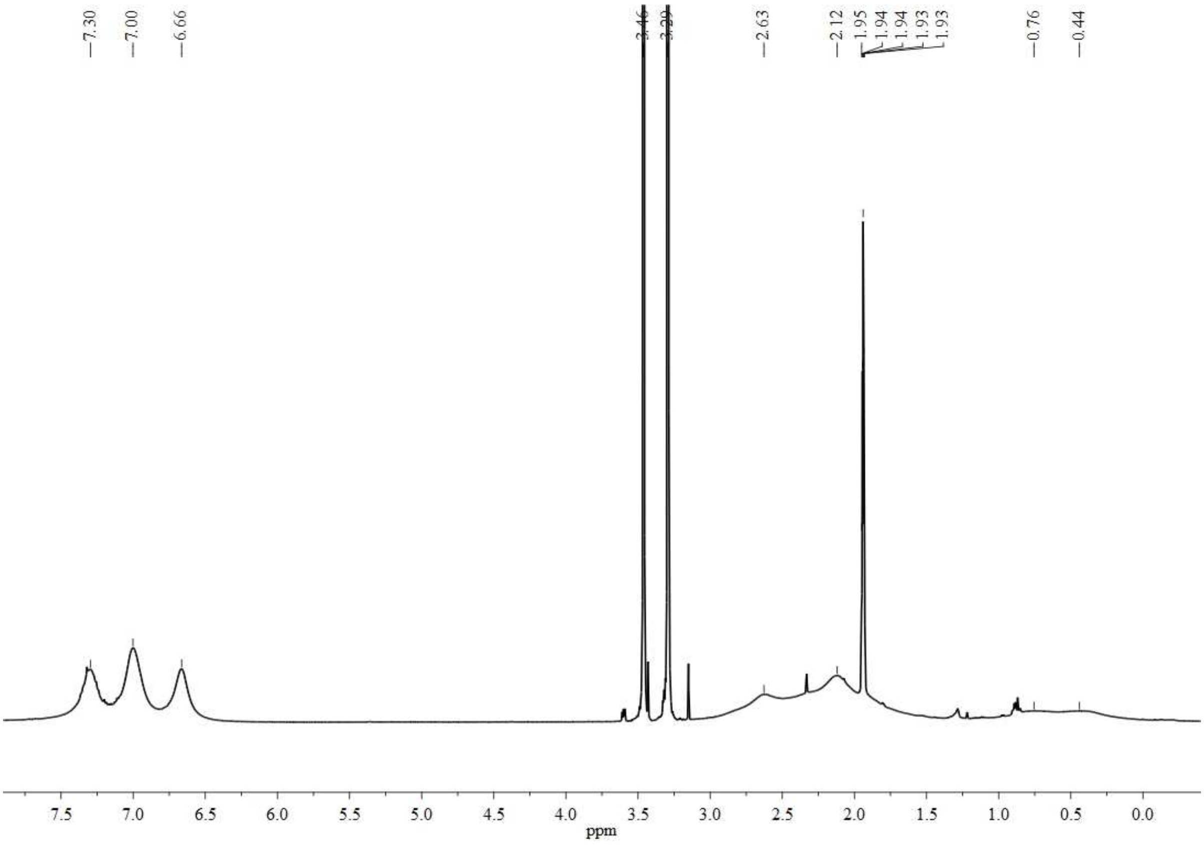

**Figure S3:** $^{13}C\{^{1}H\}$ NMR spectrum of $([Na(dme)_3]^{+})_2[\mathbf{3}]^{2-}$. Low solubility in $CD_3CN$.

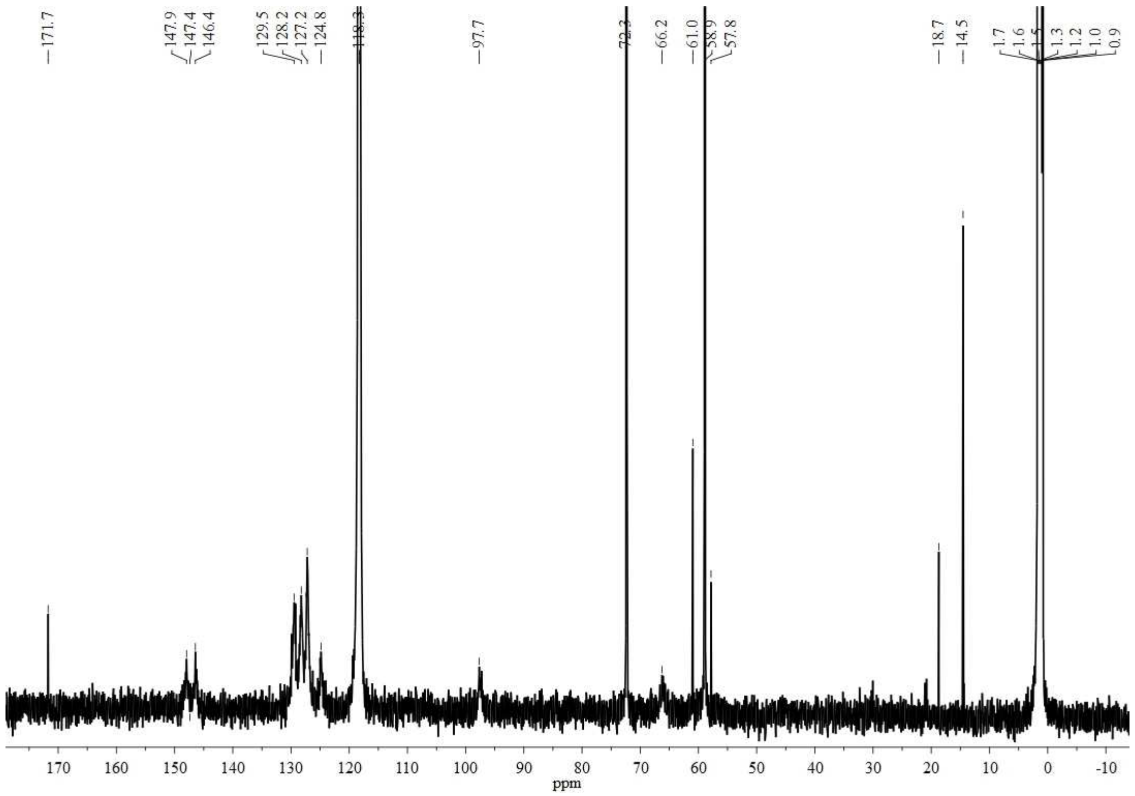


**Figure S4:** $^{11}B\{^{1}H\}$ NMR spectrum of $([Na(dme)_3]^{+})_2[\mathbf{3}]^{2-}$. Baseline correction was not applied here.

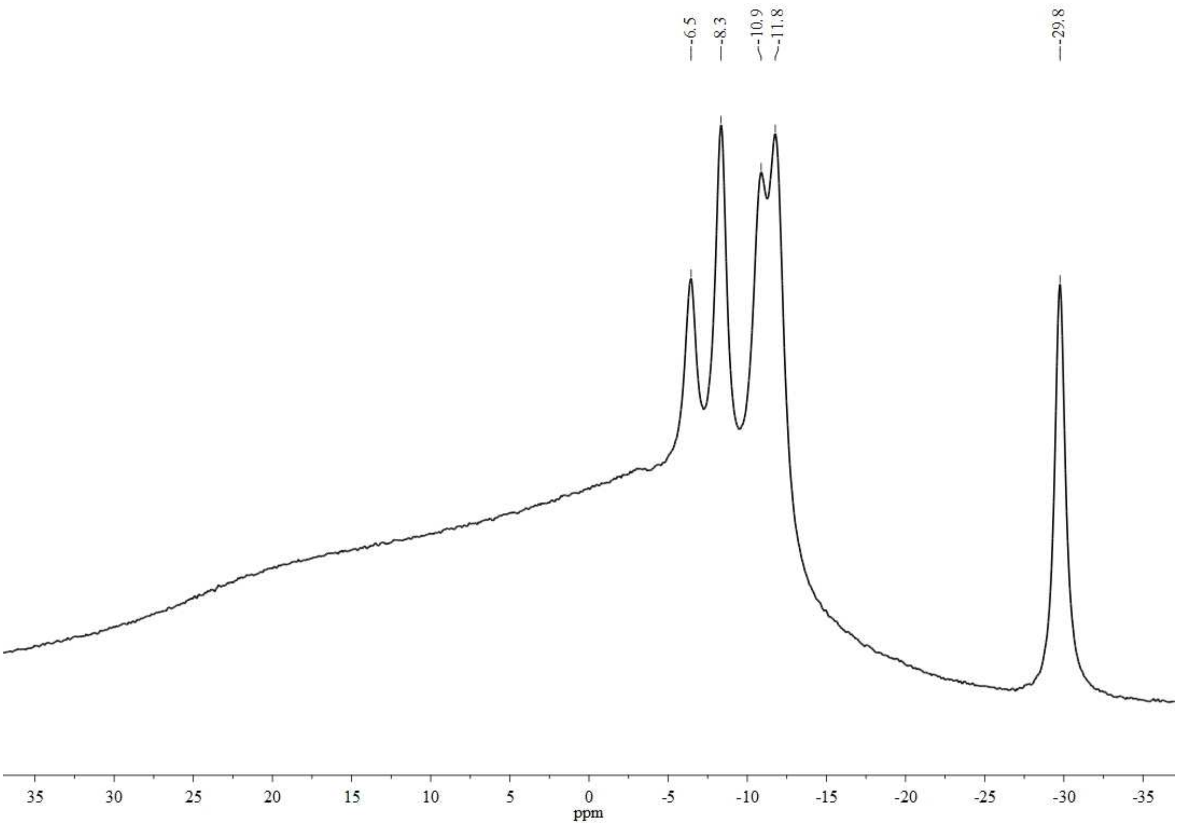

**Figure S5:** $^{11}B$ NMR spectrum of $([Na(dme)_3]^+)_2[\mathbf{3}]^{2-}$. Baseline correction was not applied here.

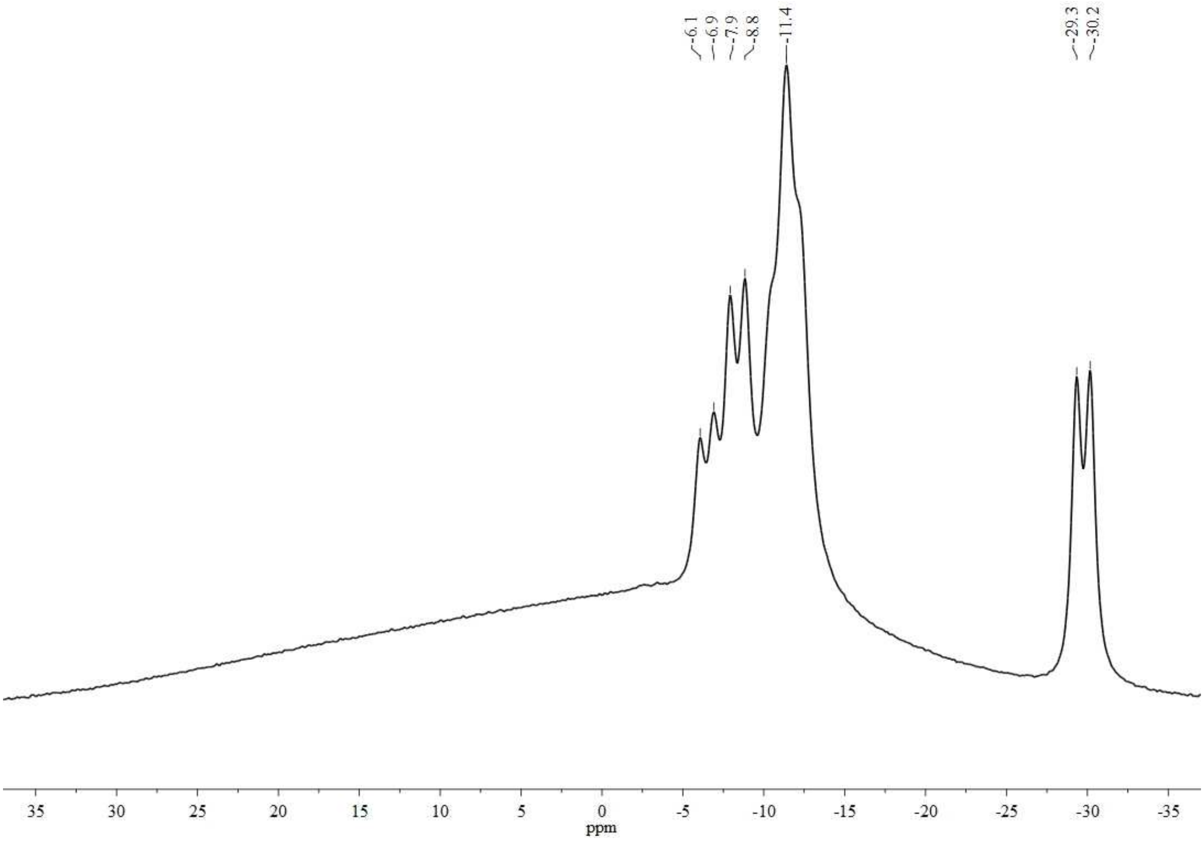


**Figure S6:** $^{1}H$ NMR spectrum of $([Na(dme)_3]^+)_2[\mathbf{4}]^{2-}$.

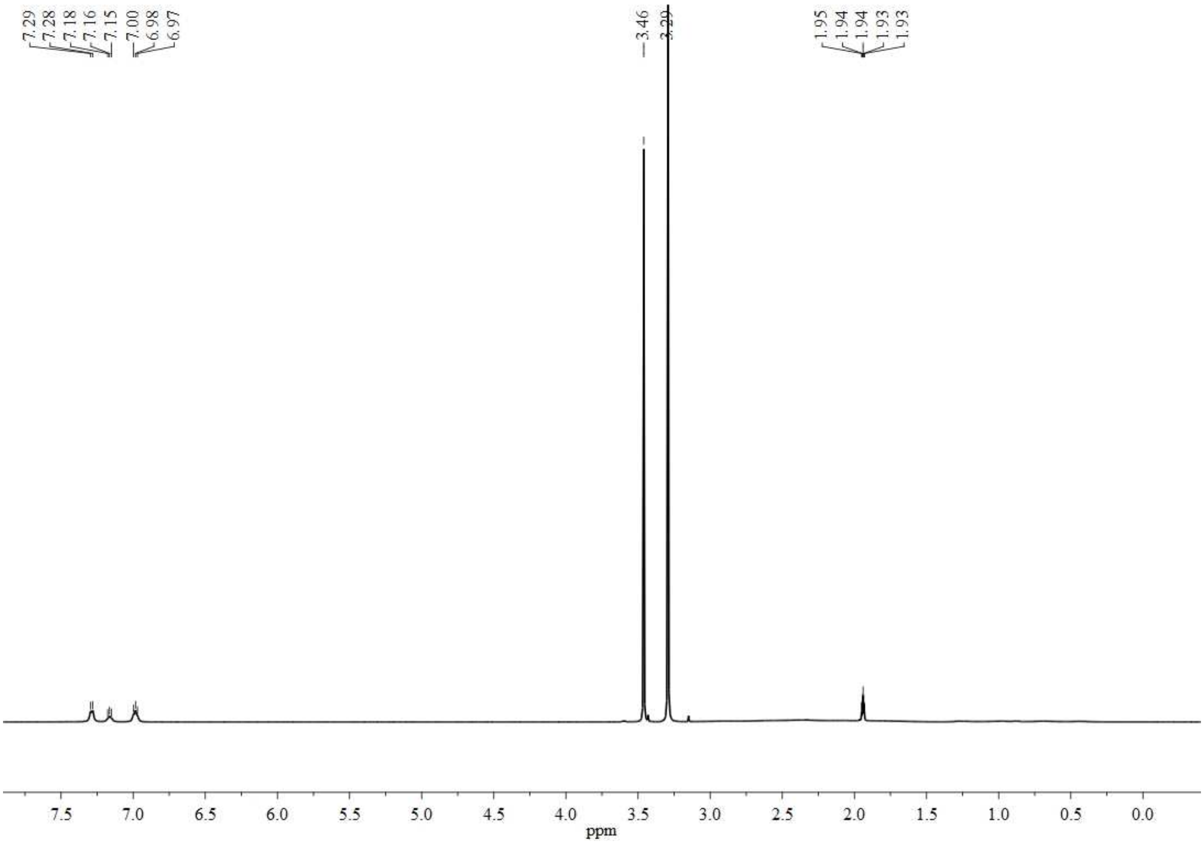

**Figure S7:** $^{1}H\{^{11}B\}$ NMR spectrum of $([Na(dme)_3]^+)_2[\mathbf{4}]^{2-}$.

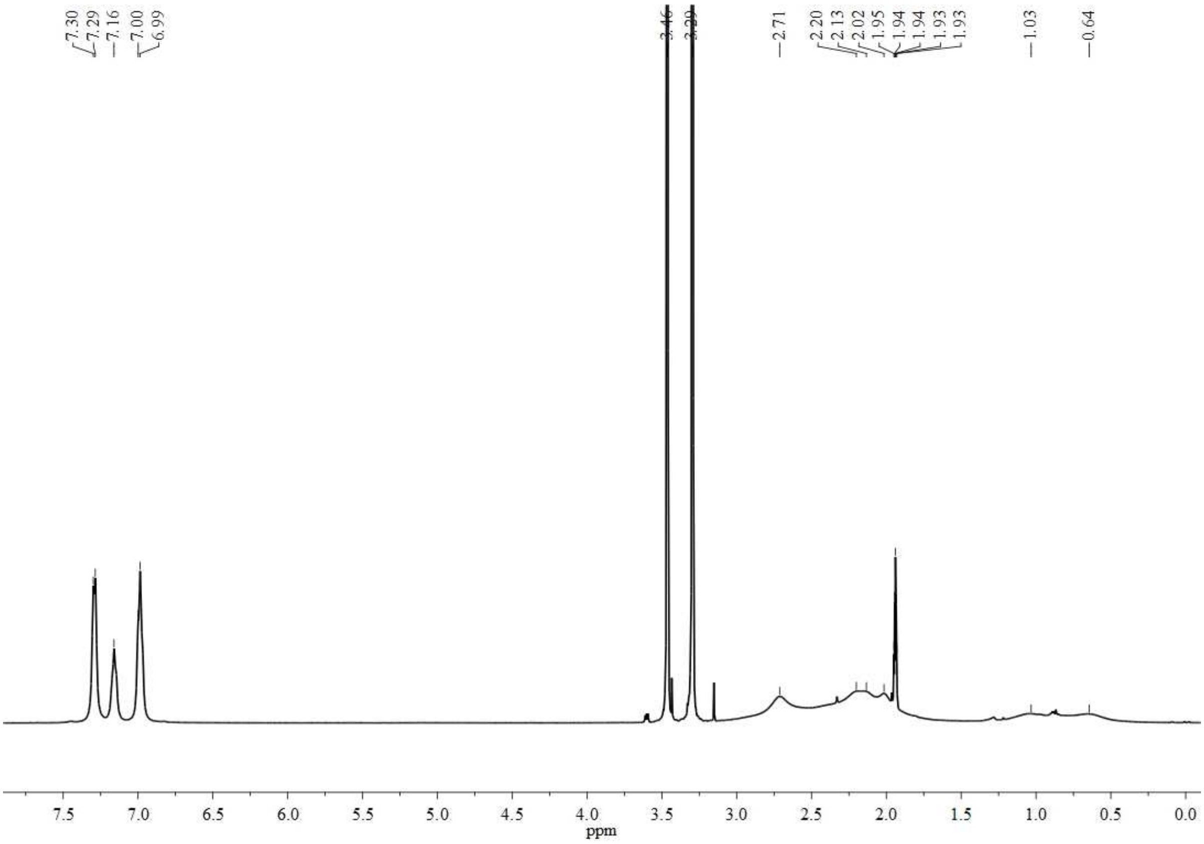


**Figure S8:** $^{13}C\{^{1}H\}$ NMR spectrum of $([Na(dme)_3]^+)_2[\mathbf{4}]^{2-}$.

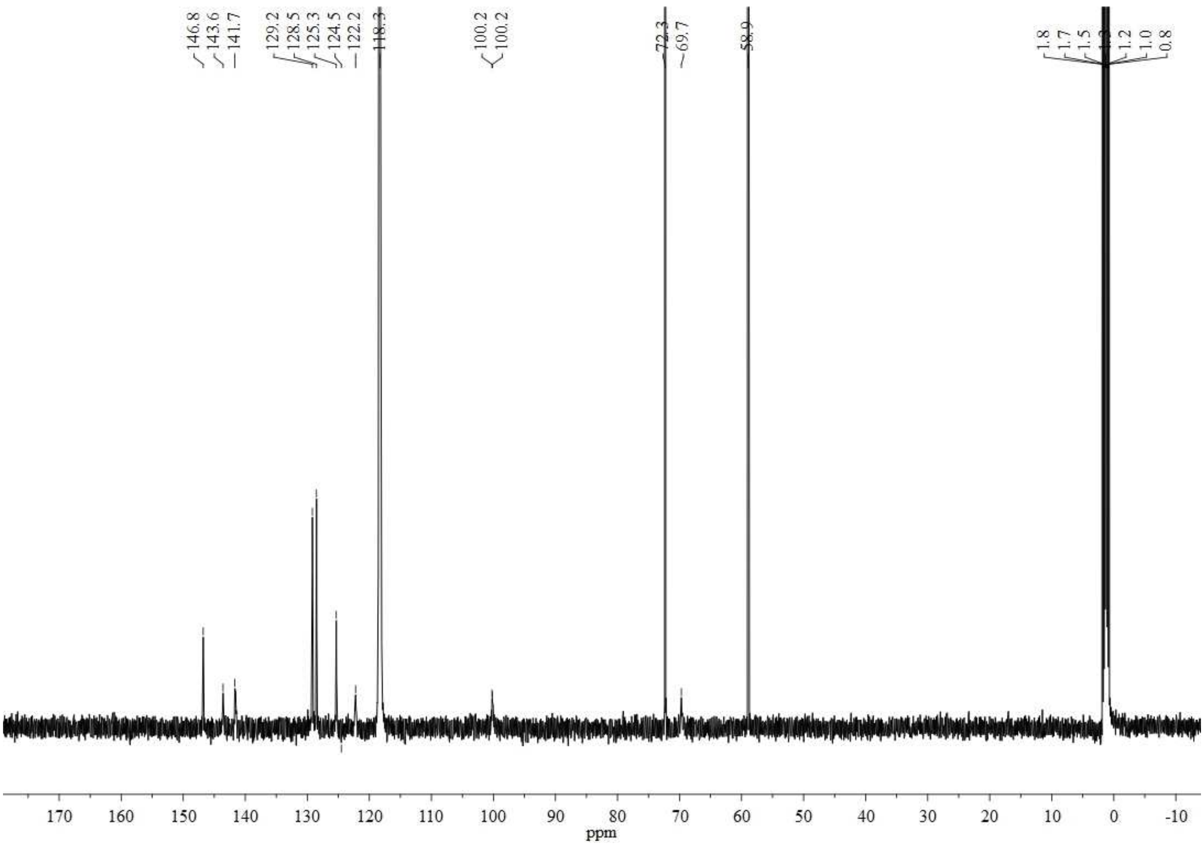

**Figure S9:** $^{11}B\{^{1}H\}$ NMR spectrum of $([Na(dme)_3]^{+})_2[\mathbf{4}]^{2-}$. Baseline correction was not applied here.

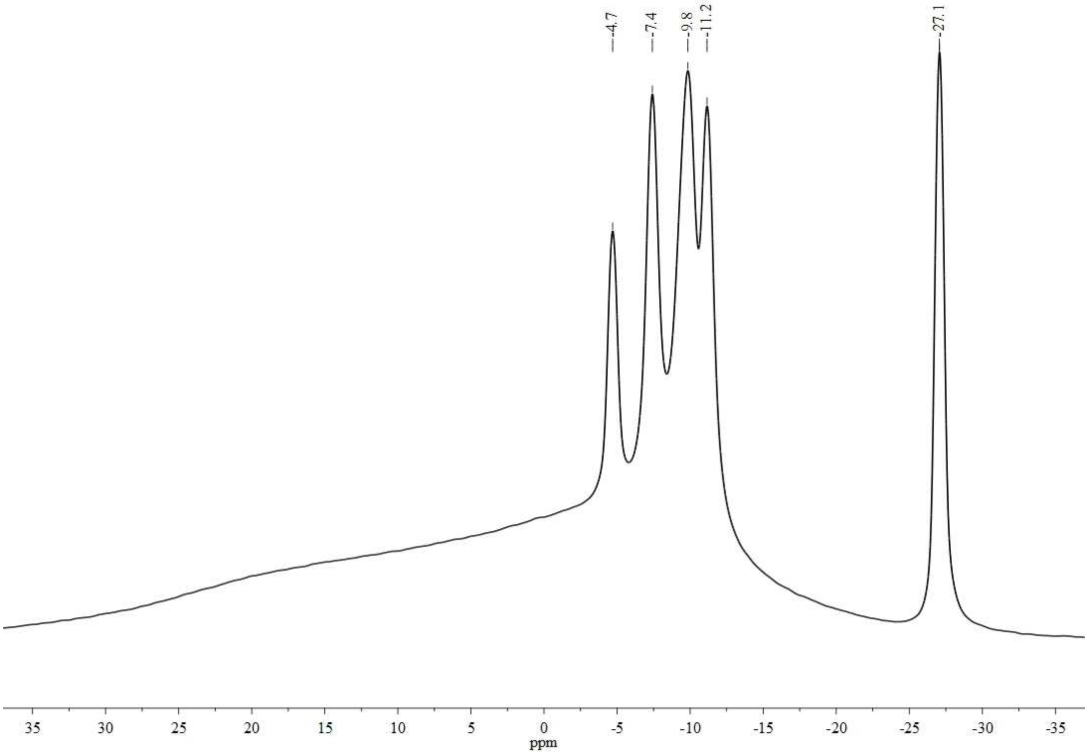


**Figure S10:** $^{11}B$ NMR spectrum of $([Na(dme)_3]^{+})_2[\mathbf{4}]^{2-}$. Baseline correction was not applied here.

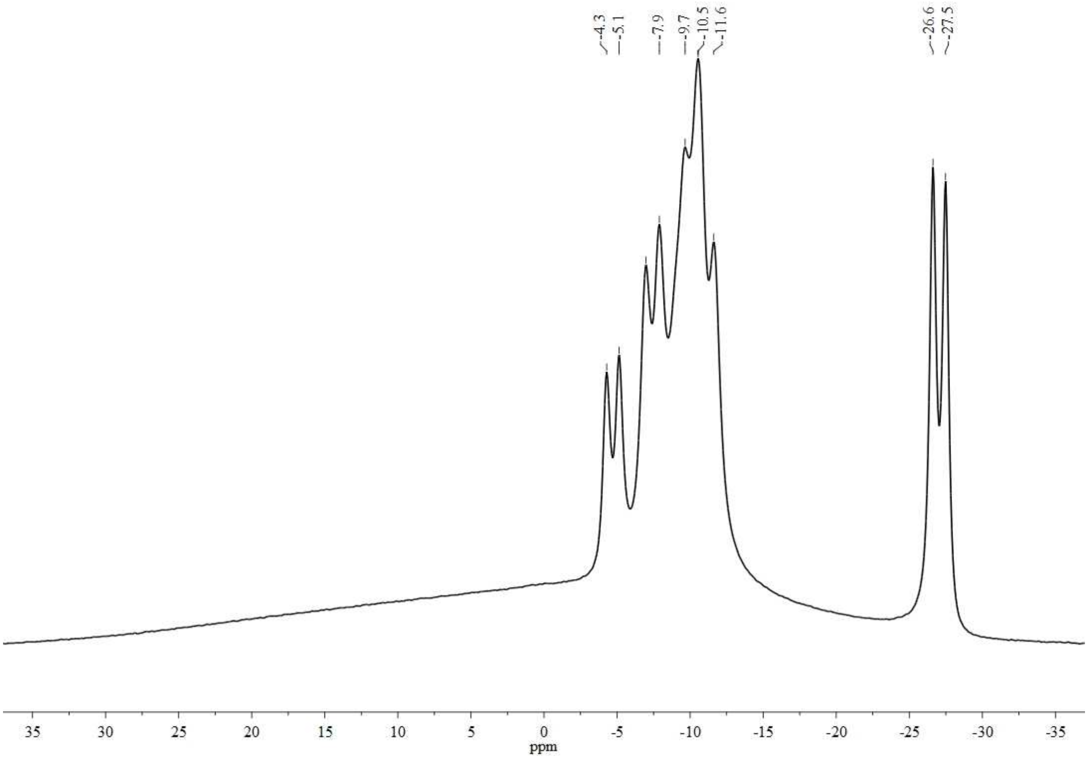

**Figure S11:** $^{19}F\{^{1}H\}$ NMR spectrum of $([Na(dme)_3]^{+})_2[\mathbf{4}]^{2-}$.

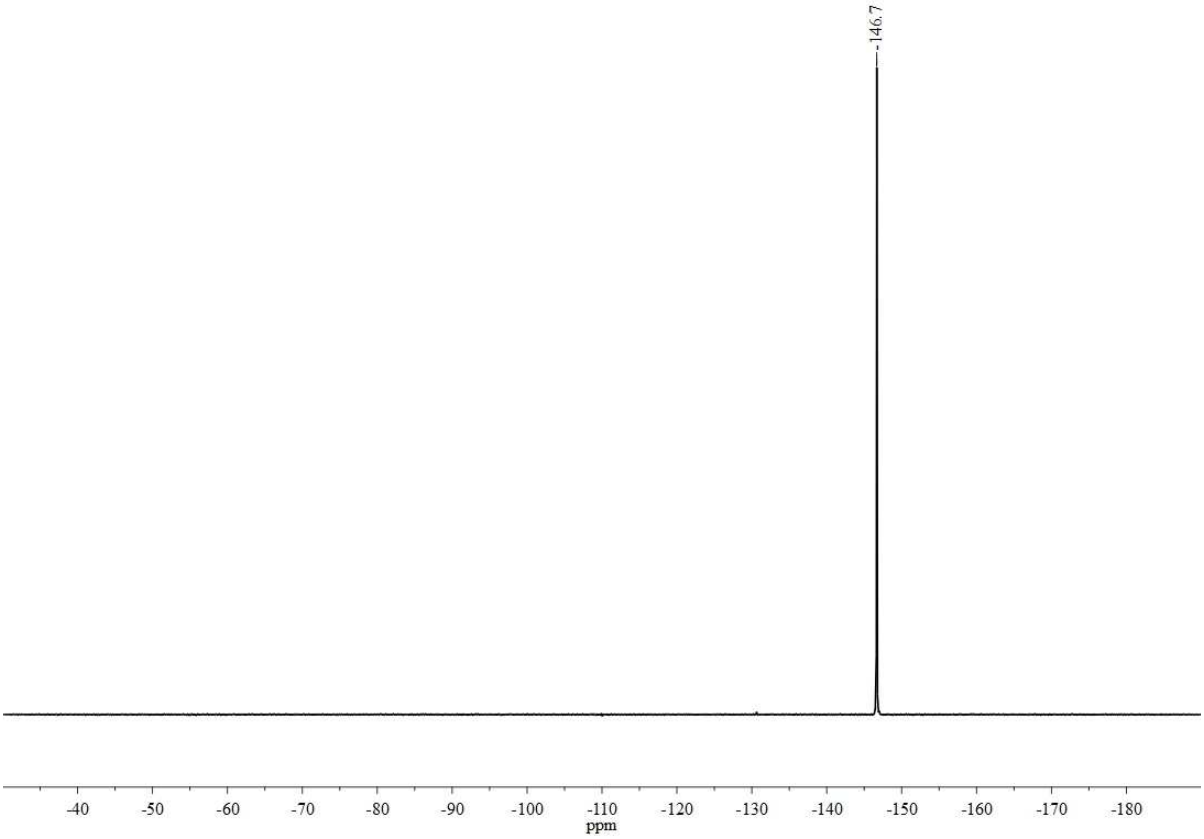

**Cyclic voltammetry**

Electrochemical measurements were carried out using an EcoChemie Autolab PG-STAT 30 potentiostat at 298 K with a glassy carbon working electrode and platinum wires as counter and reference electrodes with 0.1 M $^{n}Bu_4NPF_6$ in dichloromethane under nitrogen. Scan rates of 100 mV $s^{-1}$ and analyte concentrations of $10^{-3}$ M were used. The ferrocene/ferrocenium $FcH/FcH^+$ couple served as internal reference at 0.0 V for potential measurements.

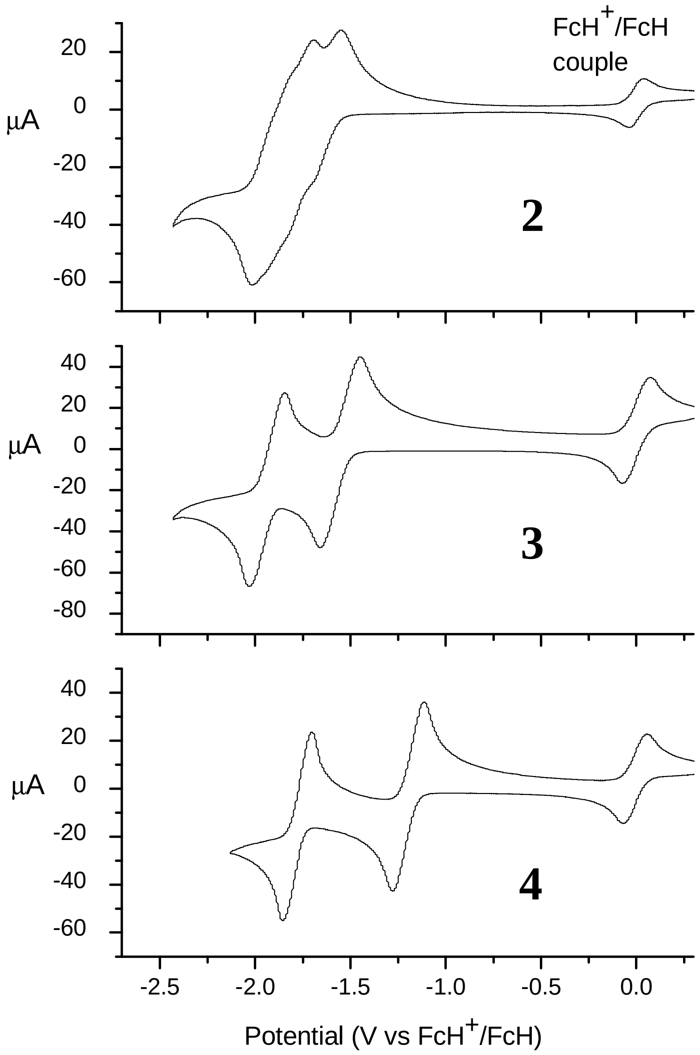


**Figure S12**. Cyclic voltammograms of **2-4** in 0.1 M $^{n}Bu_4NPF_6$ dichloromethane solutions with ferrocene as internal reference.

**Table S1**. Detailed electrochemical data for **1**,[3] **3** and **4**.

| | E(Red1) cathodic[a] (V) | E(Red1) anodic[b] (V) | $E_{1/2}$ (Red1) (V) | E(Red2) cathodic[c] (V) | E(Red2) anodic[d] (V) | $E_{1/2}$ (Red2) (V) | $E_{1/2}$ (Red1) - $E_{1/2}$ (Red2), Δ (mV) |
|---|---|---|---|---|---|---|---|
| 1e events | *0 > -1* | *-1 > 0* | 0 / -1 | *-1 > -2* | *-2 > -1* | -1 / -2 | |
| **1** | -1.90 | -1.48 | -1.69 | -1.90 | -1.80 | -1.85 | 155 |
| | | | | | | | |
| 2e events | *0 > -2* | *-2 > 0* | 0 / -2 | *-2 > -4* | *-4 > -2* | -2 / -4 | |
| **3** | -1.67 | -1.44 | -1.56 | -2.04 | -1.83 | -1.94 | 380 |
| **4** | -1.29 | -1.10 | -1.20 | -1.87 | -1.71 | -1.79 | 590 |

[a]from cathodic wave of first reduction wave

[b]from anodic wave of first reduction wave

[c]from cathodic wave of second reduction wave

[d]from anodic wave of second reduction wave

**Table S2**. Detailed electrochemical data for **2** based on square wave measurements.

| | $E_{1/2}$ (Red1) (V) | Δ (Red1-Red2) (mV) | $E_{1/2}$ (Red2) (V) | Δ (Red2-Red3) (mV) | $E_{1/2}$ (Red3) (V) | Δ (Red3-Red4) (mV) | $E_{1/2}$ (Red4) (V) |
|---|---|---|---|---|---|---|---|
| 1e events | 0 / -1 | | -1 / -2 | | -2 / -3 | | -3 / -4 |
| **2** | -1.60 | 150 | -1.75 | 110 | -1.86 | 90 | -1.95 |

**Figure S13.** Square wave voltammetry traces for **2** from negative to positive potentials and vice versa. All four reversible one-electron reduction waves are evident.

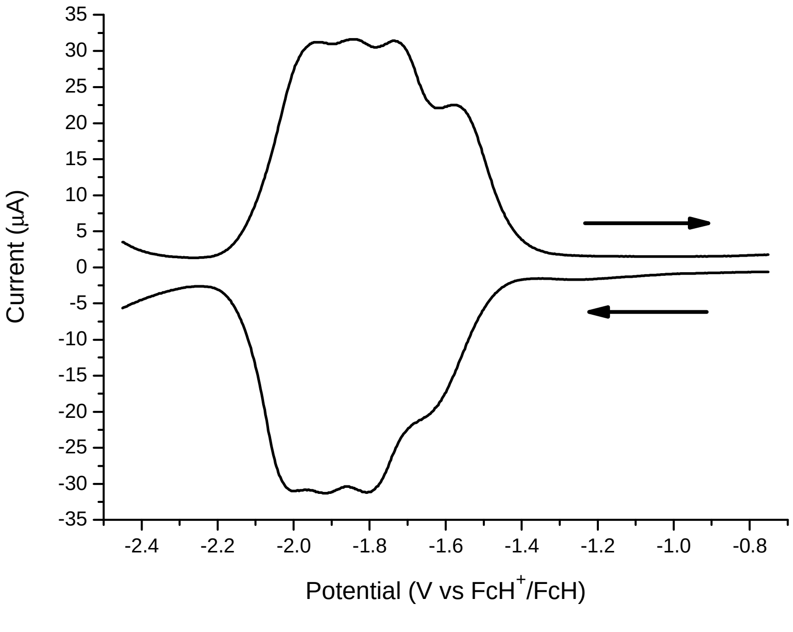

## Crystallography

Single crystals were coated with a layer of hydrocarbon oil and attached to a glass fiber. Crystallographic data were collected with a Bruker AXS X8 Prospector Ultra with APEX II for $([Na(dme)_3]^+)_2[\mathbf{3}]^{2-}$ with Cu-$K_\alpha$ radiation (multilayer optic, $\lambda$ = 1.54178 Å) and a Nonius Kappa CCD diffractometer with Mo-$K_\alpha$ radiation (graphite monochromator, $\lambda$ = 0.71073 Å) at 100(2) K for $([Na(dme)_3]^+)_2[\mathbf{4}]^{2-}$. Crystallographic programs used for structure solution and refinement were from SHELX-97.[4] The structures were solved by direct methods and were refined by using full-matrix least squares on $F^2$ of all unique reflections with anisotropic thermal parameters for all non-hydrogen atoms. The hydrogen atoms bonded to boron atoms were refined isotropically, all other hydrogen atoms were refined using a riding model with U(H) = 1.2 $U_{eq}$. Crystallographic data for the compounds are listed in Table S3. CCDC-972505 $([Na(dme)_3]^+)_2[\mathbf{3}]^{2-}$ and CCDC-972506 $([Na(dme)_3]^+)_2[\mathbf{4}]^{2-}$, contain the supplementary crystallographic data for this paper. These data can be obtained free of charge from the Cambridge Crystallographic Data Centre via www.ccdc.cam.ac.uk/data_request/cif.

**Figure S14.** Molecular structures of $([Na(dme)_3]^+)_2[\mathbf{3}]^{2-}$ and $([Na(dme)_3]^+)_2[\mathbf{4}]^{2-}$.

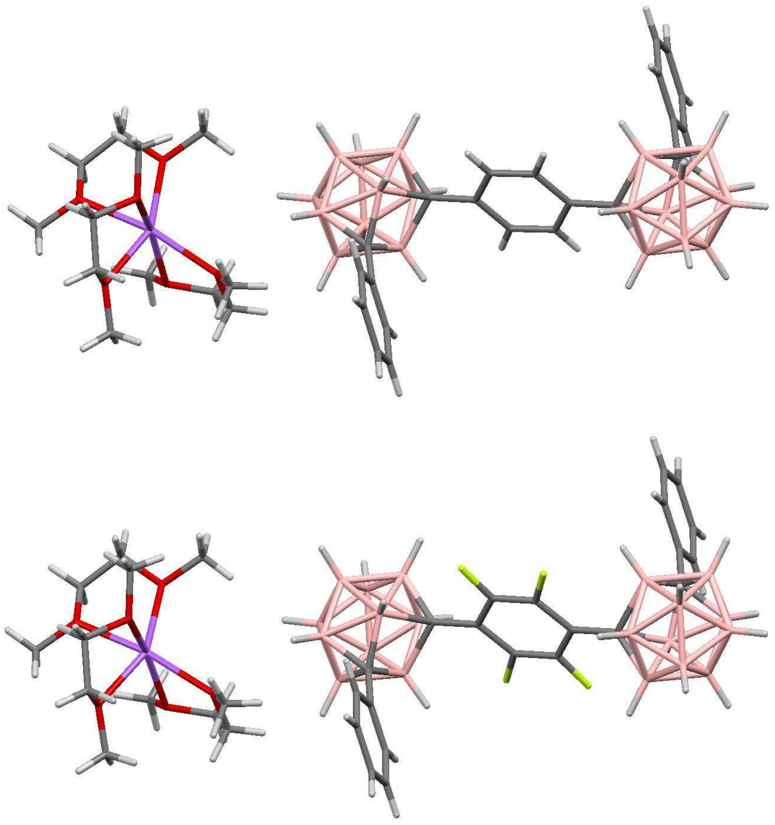

**Table S3.** Crystallographic data for compounds $([Na(dme)_3]^+)_2[\mathbf{3}]^{2-}$ and $([Na(dme)_3]^+)_2[\mathbf{4}]^{2-}$.

| | | |
|---|---|---|
| Compound | $([Na(dme)_3]^+)_2[\mathbf{3}]^{2-}$ (CCDC-972505) | $([Na(dme)_3]^+)_2[\mathbf{4}]^{2-}$ (CCDC-972506) |
| Diffractometer | Bruker AXS X8 Prospector Ultra with APEX II | Nonius Kappa CCD |
| Temperature [K] | 100(2) | 100(2) |
| Wavelength [Å] | 1.54178 | 0.71073 |
| Formula | $C_{22}H_{34}B_{20}$, $2(C_{12}H_{30}NaO_6)$ | $C_{22}H_{30}B_{20}F_4$, $2(C_{12}H_{30}NaO_6)$ |
| $M_r$ [g $mol^{-1}$] | 1101.39 | 1173.36 |
| Crystal system, space group | Monoclinic $P2_1/c$ | Monoclinic $P2_1/c$ |
| Unit cell dimensions | a = 12.1461(3) Å<br>b = 10.2658(3) Å<br>c = 25.8117(6) Å<br>β = 95.4480(10)° | a = 12.2218(2) Å<br>b = 10.31520(10) Å<br>c = 26.2012(3) Å<br>β = 95.2167(6)° |
| Volume [$Å^3$] | 3203.91(14) | 3289.51(7) |
| Z, calc. density [$Mg/m^3$] | 2, 1.142 | 2, 1.185 |
| Absorption coefficient [$mm^{-1}$] | 0.684 | 0.094 |
| F(000) | 1176 | 1240 |
| Crystal size [$mm^3$], color and habit | 0.31 x 0.30 x 0.13, dark green plate | 0.30 x 0.22 x 0.18, dark green fragment |
| θ range [°] | 3.44 - 69.8 | 2.94 - 27.5 |
| Reflections collected / unique | 38779 / 5860 | 90453 / 7540 |
| R(int) | 0.0200 | 0.144 |
| Data Completeness | 97.0% | 99.7% |
| Data / restraints / parameters | 5860 / 0 / 407 | 7540 / 0 / 425 |
| Goodness-of-fit ($F^2$) | 1.024 | 1.035 |
| Final *R1*, *wR2* [$I>2\sigma(I)$] | 0.0303, 0.0803 [5671] | 0.0337, 0.0870 [5474] |
| *R1*, *wR2* (all data) | 0.0311, 0.0811 | 0.0554, 0.0945 |
| Largest diff. peak and hole [$eÅ^{-3}$] | 0.208 and -0.216 | 0.246 and -0.196 |

**Computations**

All computations were carried out with the Gaussian 03 package.[5] The geometries of **1**, [**1**]$^-$, **3**, [**3**]$^{2-}$, **4** and [**4**]$^{2-}$ were fully optimized at the ground states ($S_0$) with the B3LYP functional[6] with no symmetry constraints using the 6-31G* basis set[7] for all atoms. Frequency calculations on these optimized geometries revealed no imaginary frequencies. Natural Population Analyses (NPA) were carried out on the optimized geometries using the Gaussian NBO 3.1 program for Wiberg bond index and charge data.

**Table S4.** Computed bond indices (BI) and charges on optimized geometries of **1**, [**1**]$^-$, **3**, [**3**]$^{2-}$, **4** and [**4**]$^{2-}$ at B3LYP/6-31G*.

| | **1** | [**1**]$^-$ | **3** | [**3**]$^{2-}$ | **4** | [**4**]$^{2-}$ |
|---|---|---|---|---|---|---|
| Bond distance | | | | | | |
| C1-C2 (Å) | 1.76 | 2.39 | 1.76 | 2.42 | 1.78 | 2.44 |
| Wiberg BI | | | | | | |
| C1-C2 | 0.59 | 0.06 | 0.60 | 0.06 | 0.59 | 0.06 |
| C1-Ph | 1.00 | 1.05 | 1.00 | 1.03 | 1.00 | 1.03 |
| C2-C3 | | | 1.00 | 1.22 | 1.00 | 1.24 |
| C3-C4 | | | 1.39 | 1.23 | 1.34 | 1.24 |
| C4-C5’ | | | 1.45 | 1.60 | 1.35 | 1.51 |
| Charges | | | | | | |
| -$C_2B_{10}H_{10}$- | -0.23 | -0.88 | -0.22 | -0.99 | -0.20 | -0.96 |
| -$C_6H_5$ | 0.12 | -0.06 | 0.12 | -0.02 | 0.12 | 0.02 |
| -$C_6X_4$- | | | 0.20 | 0.06 | 0.15 | -0.12 |

Calculated $^{11}$B, $^{13}$C and $^1$H NMR chemical shifts obtained at the GIAO[8]-B3LYP/6-31G*//B3LYP/6-31G* level on the optimized geometries were referenced to $BF_3{\cdot}OEt_2$ for $^{11}$B: $\delta\ (^{11}B) = 110.7 - \sigma(^{11}B)$ and referenced to TMS for $^{13}$C: $\delta\ (^{13}C) = 195.0 - \sigma(^{13}C)$. Table S5 shows computed and observed NMR data for [**3**]$^{2-}$ and [**4**]$^{2-}$ which are in good agreement and Figure S15 shows the observed $^{11}B\{^1H\}$ NMR peaks assigned for [**3**]$^{2-}$.

**Table S5:** $^{11}$B and $^{13}$C GIAO-NMR chemical shifts for [**3**]$^{2-}$ and [**4**]$^{2-}$ and comparison with observed values.

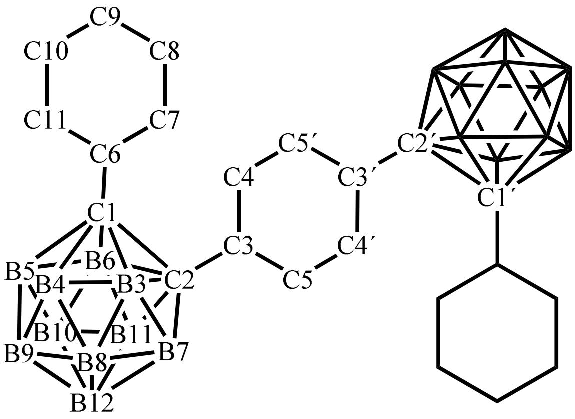


| [**3**]$^{2-}$ Calc. | Peak assignment | ([Na(dme)$_3$]$^+$)$_2$[**3**]$^{2-}$ Observed | [**4**]$^{2-}$ Calc. | Peak assignment | ([Na(dme)$_3$]$^+$)$_2$[**4**]$^{2-}$ Observed |
|---|---|---|---|---|---|
| $^{11}$B NMR | | | | | |
| –29.6 | B8,10 | –29.8 (2) | –27.1 | B8,10 | –27.1 (2) |
| –11.3 | B3,6 | –11.8 (3) | –10.6 | B7,11 | –11.2 (2) |
| -11.2 | B9 | | –9.7 | B3,6 | –9.8 (3) |
| –10.9 | B7,11 | –10.9 (2) | –9.1 | B9 | |
| –7.8 | B4,5 | –8.3 (2) | –7.1 | B4,5 | –7.4 (2) |
| –4.5 | B12 | –6.5 (1) | –2.9 | B12 | –4.7 (1) |
| $^{13}$C NMR | | | | | |
| 64.9 | C1 | 66.2 | 68.6 | C1 | 69.7 |
| 104.9 | C2 | 97.7 | 106.3 | C2 | 100.2 |
| 117.8 | C9 | 124.9 | 119.7 | C9 | 125.3 |
| 123.2 | C8,10 | 127.2 | 123.5 | C8,10 | 128.5 |
| 126.2 | C4,5 | 128.2 | 127.9 | C3 | 122.2 |
| 128.9 | C7,11 | 129.5 | 129.3 | C7,11 | 129.2 |
| 149.0 | C3 | 146.4 | 144.4 | C4,5 | 142.6 |
| 151.9 | C6 | 147.9 | 151.5 | C6 | 146.8 |

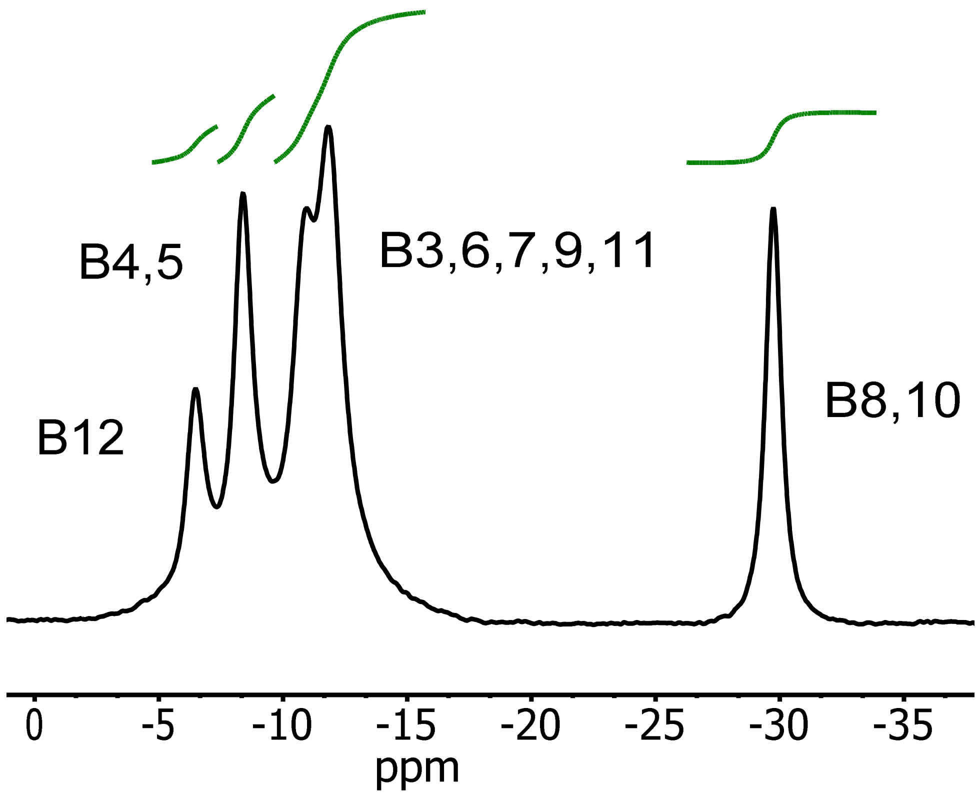


**Figure S15.** Observed $^{11}B\{^{1}H\}$ NMR spectrum for $([Na(dme)_3]^{+})_2[\mathbf{3}]^{2-}$ with peak assignment based on GIAO-NMR predictions.

Cartesian coordinates for optimized geometries of [**3**]$^{2-}$ and [**4**]$^{2-}$

[**3**]$^{2-}$

| | | | |
|---|---|---|---|
| C | 0.00000 | 0.00000 | 0.00000 |
| C | -2.51845 | -1.14883 | 0.84143 |
| C | -1.23472 | 0.31140 | -0.65497 |
| C | -2.43028 | -0.22510 | -0.24925 |
| C | -0.08806 | -0.92365 | 1.09066 |
| C | -1.28370 | -1.46015 | 1.49644 |
| C | 1.23643 | 0.56972 | -0.40906 |
| C | -3.75485 | -1.71847 | 1.25053 |
| H | -1.21499 | 1.00101 | -1.49355 |
| H | -3.34319 | 0.04730 | -0.77040 |
| H | 0.82479 | -1.19619 | 1.61190 |
| H | -1.30330 | -2.14980 | 2.33504 |
| C | -5.18301 | -0.69563 | 2.91281 |
| B | -4.12580 | -2.01805 | 2.99154 |
| B | -4.08283 | -3.26765 | 1.63389 |
| B | -4.94256 | -2.34107 | 0.32526 |
| B | -5.28802 | -0.76569 | 1.22277 |
| B | -5.85994 | -1.99271 | 3.67584 |
| B | -5.22075 | -3.47445 | 2.96592 |
| B | -5.79650 | -3.59073 | 1.26633 |
| B | -6.57741 | -2.01254 | 0.90116 |
| B | -6.70058 | -1.08702 | 2.39645 |
| B | -6.83769 | -2.84978 | 2.47496 |
| H | -3.20166 | -1.96071 | 3.73960 |
| H | -3.19866 | -4.05006 | 1.46667 |
| H | -4.71616 | -2.41469 | -0.84314 |
| H | -5.25753 | 0.25464 | 0.61058 |
| H | -6.09392 | -1.88667 | 4.84143 |
| H | -5.09215 | -4.44405 | 3.65420 |
| H | -6.10445 | -4.61993 | 0.73995 |
| H | -7.44072 | -1.91318 | 0.07959 |
| H | -7.57144 | -0.29465 | 2.59285 |
| H | -7.86999 | -3.36820 | 2.78623 |
| C | 2.66496 | -0.45342 | -2.07097 |
| B | 2.76970 | -0.38264 | -0.38079 |
| B | 2.42388 | 1.19297 | 0.51601 |
| B | 1.56415 | 2.11878 | -0.79318 |
| B | 1.60756 | 0.86868 | -2.15034 |
| B | 4.18242 | -0.06164 | -1.55440 |
| B | 4.05887 | 0.86455 | -0.05950 |
| B | 3.27770 | 2.44239 | -0.42543 |
| B | 2.70220 | 2.32530 | -2.12520 |
| B | 3.34179 | 0.84350 | -2.83432 |
| B | 4.31918 | 1.70113 | -1.63363 |
| H | 2.73945 | -1.40278 | 0.23179 |
| H | 2.19737 | 1.26709 | 1.68441 |
| H | 0.67985 | 2.90108 | -0.62635 |
| H | 0.68353 | 0.81085 | -2.89842 |
| H | 5.05339 | -0.85395 | -1.75027 |
| H | 4.92214 | 0.76573 | 0.76220 |
| H | 3.58537 | 3.47190 | 0.10051 |
| H | 2.57352 | 3.29461 | -2.81378 |
| H | 3.57601 | 0.73691 | -3.99991 |
| H | 5.35151 | 2.21961 | -1.94503 |
| C | -4.88766 | 0.57410 | 3.61780 |
| C | -4.35418 | 3.00264 | 4.98657 |
| C | -5.05655 | 3.00675 | 3.77908 |

| | | | |
|---|---|---|---|
| C | -5.31818 | 1.81479 | 3.10684 |
| C | -3.91993 | 1.78189 | 5.50905 |
| C | -4.18126 | 0.58981 | 4.83708 |
| H | -4.14331 | 3.93416 | 5.50759 |
| H | -5.86340 | 1.83570 | 2.16899 |
| H | -5.40096 | 3.94710 | 3.35239 |
| H | -3.83544 | -0.34948 | 5.25554 |
| H | -3.36772 | 1.75632 | 6.44679 |
| C | 2.36999 | -1.72356 | -2.77538 |
| C | 1.83716 | -4.15271 | -4.14334 |
| C | 1.40273 | -2.93237 | -4.66627 |
| C | 1.66373 | -1.73994 | -3.99481 |
| C | 2.53951 | -4.15620 | -2.93568 |
| C | 2.80075 | -2.96402 | -2.26386 |
| H | 1.62657 | -5.08453 | -4.66393 |
| H | 1.31777 | -0.80076 | -4.41366 |
| H | 0.85069 | -2.90717 | -5.60411 |
| H | 3.34584 | -2.98430 | -1.32603 |
| H | 2.88397 | -5.09631 | -2.50856 |

$[\mathbf{4}]^{2-}$

| | | | |
|---|---|---|---|
| C | 0.00000 | 0.00000 | 0.00000 |
| C | -2.40860 | -1.29537 | 1.15461 |
| C | 0.01431 | -0.79730 | 1.19119 |
| C | -1.09381 | -1.40854 | 1.71444 |
| C | -1.31485 | 0.11326 | -0.55974 |
| C | -2.42288 | -0.49798 | -0.03655 |
| C | 1.15527 | 0.58232 | -0.57408 |
| C | -3.56385 | -1.87760 | 1.72872 |
| F | 1.17895 | -0.97105 | 1.85374 |
| F | -0.91267 | -2.15684 | 2.82473 |
| F | -1.49599 | 0.86151 | -1.67019 |
| F | -3.58759 | -0.32414 | -0.69901 |
| C | -4.95211 | -0.73562 | 3.37578 |
| B | -3.80717 | -1.96185 | 3.55190 |
| B | -3.76076 | -3.36689 | 2.34642 |
| B | -4.78209 | -2.66723 | 1.00037 |
| B | -5.19026 | -1.01445 | 1.72925 |
| B | -5.48334 | -1.95734 | 4.35024 |
| B | -4.78864 | -3.47315 | 3.77772 |
| B | -5.47171 | -3.83881 | 2.15700 |
| B | -6.39087 | -2.37550 | 1.66604 |
| B | -6.47516 | -1.27779 | 3.04308 |
| B | -6.47689 | -3.02393 | 3.34332 |
| H | -2.85065 | -1.74124 | 4.21481 |
| H | -2.85082 | -4.12179 | 2.20896 |
| H | -4.64933 | -2.88967 | -0.16132 |
| H | -5.28100 | -0.07625 | 1.01179 |
| H | -5.63578 | -1.71580 | 5.50867 |
| H | -4.53794 | -4.33898 | 4.56251 |
| H | -5.73760 | -4.94372 | 1.78438 |
| H | -7.31179 | -2.43876 | 0.90696 |
| H | -7.38162 | -0.51977 | 3.20778 |
| H | -7.44358 | -3.56288 | 3.79668 |
| C | 2.54309 | -0.55935 | -2.22173 |
| B | 2.78163 | -0.28095 | -0.57517 |
| B | 2.37374 | 1.37169 | 0.15424 |
| B | 1.35210 | 2.07172 | -1.19139 |
| B | 1.39820 | 0.66701 | -2.39728 |

| | | | |
|---|---|---|---|
| B | 4.06622 | -0.01724 | -1.88925 |
| B | 3.98237 | 1.08006 | -0.51195 |
| B | 3.06319 | 2.54349 | -1.00228 |
| B | 2.37967 | 2.17835 | -2.62294 |
| B | 3.07407 | 0.66259 | -3.19602 |
| B | 4.06803 | 1.72884 | -2.18904 |
| H | 2.87251 | -1.21930 | 0.14197 |
| H | 2.24132 | 1.59379 | 1.31604 |
| H | 0.44222 | 2.82669 | -1.05353 |
| H | 0.44149 | 0.44658 | -3.05995 |
| H | 4.97270 | -0.77533 | -2.05442 |
| H | 4.90346 | 1.14310 | 0.24702 |
| H | 3.32919 | 3.64832 | -0.62951 |
| H | 2.12886 | 3.04437 | -3.40755 |
| H | 3.22628 | 0.42125 | -4.35458 |
| H | 5.03461 | 2.26787 | -2.64255 |
| C | -4.69680 | 0.63392 | 3.89391 |
| C | -4.24219 | 3.24390 | 4.90568 |
| C | -5.02830 | 3.05057 | 3.76764 |
| C | -5.25133 | 1.76818 | 3.27020 |
| C | -3.68495 | 2.13034 | 5.53810 |
| C | -3.90808 | 0.84786 | 5.04069 |
| H | -4.06168 | 4.24580 | 5.28954 |
| H | -5.86168 | 1.63486 | 2.38340 |
| H | -5.46867 | 3.90528 | 3.25760 |
| H | -3.46559 | -0.00671 | 5.54145 |
| H | -3.06616 | 2.25931 | 6.42411 |
| C | 2.28755 | -1.92874 | -2.74015 |
| C | 1.83255 | -4.53846 | -3.75244 |
| C | 1.27525 | -3.42469 | -4.38444 |
| C | 1.49855 | -2.14233 | -3.88680 |
| C | 2.61894 | -4.34547 | -2.61452 |
| C | 2.84224 | -3.06311 | -2.11689 |
| H | 1.65187 | -5.54023 | -4.13652 |
| H | 1.05606 | -1.28765 | -4.38723 |
| H | 0.65629 | -3.55344 | -5.27034 |
| H | 3.45271 | -2.93015 | -1.23009 |
| H | 3.05944 | -5.20034 | -2.10480 |

**References**


[1] a) T. G. Hibbert, N. D. Tinker, K. Wade, 'Metallacarboranes' *US Patent* 6492570 **1998**; b) T. G. Hibbert, 'New chemistry of icosahedral carborane derivatives', Ph.D. Thesis **1997**; Available at http://etheses.dur.ac.uk/1713/.

[2] L. I. Zakharkin, V. N. Lebedev, *Bull. Chem. Soc. USSR, Div. Chem. Sci.* **1972**, 2273-2275. (Engl. Transl.)

[3] L. Weber, J. Kahlert, R. Brockhinke, L. Böhling, J. Halama, A. Brockhinke, H.-G. Stammler, B. Neumann, C. Nervi, R. A. Harder, M. A. Fox, *Dalton Trans.* **2013**, *42*, 10982-10996.

[4] G. M. Sheldrick, *Acta Cryst*. **2008**, *A64*, 112-122.

[5] Gaussian 03, Revision E.01, M. J. Frisch, G. W. Trucks, H. B. Schlegel, G. E. Scuseria, M. A. Robb, J. R. Cheeseman, J. A. Montgomery, Jr., T. Vreven, K. N. Kudin, J. C. Burant, J. M. Millam, S. S. Iyengar, J. Tomasi, V. Barone, B. Mennucci, M. Cossi, G. Scalmani, N. Rega, G. A. Petersson, H. Nakatsuji, M. Hada, M. Ehara, K. Toyota, R. Fukuda, J. Hasegawa, M. Ishida, T. Nakajima, Y. Honda, O. Kitao, H. Nakai, M. Klene, X. Li, J. E. Knox, H. P. Hratchian, J. B. Cross, V. Bakken, C. Adamo, J. Jaramillo, R. Gomperts, R. E. Stratmann, O. Yazyev, A. J. Austin, R. Cammi, C. Pomelli, J. W. Ochterski, P. Y. Ayala, K. Morokuma, G. A. Voth, P. Salvador, J. J. Dannenberg, V. G. Zakrzewski, S. Dapprich, A. D. Daniels, M. C. Strain, O. Farkas, D. K. Malick, A. D. Rabuck, K. Raghavachari, J. B. Foresman, J. V. Ortiz, Q. Cui, A. G. Baboul, S. Clifford, J. Cioslowski, B. B. Stefanov, G. Liu, A. Liashenko, P. Piskorz, I. Komaromi, R. L. Martin, D. J. Fox, T. Keith, M. A. Al-Laham, C. Y. Peng, A. Nanayakkara, M. Challacombe, P. M. W. Gill, B. Johnson, W. Chen, M. W. Wong, C. Gonzalez, J. A. Pople, Gaussian, Inc., Wallingford CT **2004**.

[6] a) A. D. Becke, *J. Chem. Phys.* **1993**, *98*, 5648-5652; b) C. Lee, W. Yang, R. G. Parr, *Phys. Rev. B* **1988**, *37*, 785-789.

[7] a) G. A. Petersson, M. A. Al-Laham, *J. Chem. Phys.* **1991**, *94*, 6081-6090; b) G. A. Petersson, A. Bennett, T. G. Tensfeldt, M. A. Al-Laham, W. A. Shirley, J. Mantzaris, *J. Chem. Phys.* **1988**, *89*, 2193-2218.

[8] a) R. Ditchfield, *Mol. Phys.* **1974**, *27*, 789-807; b) C. M. Rohling, L. C. Allen, R. Ditchfield, *Chem. Phys.* **1984**, *87*, 9-15; c) K. Wolinski, J. F. Hinton, P. Pulay, *J. Am. Chem. Soc.* **1990**, *112*, 8251-8260.
